# Preeclampsia Induced Liver Dysfunction Complicated by Disseminated Intravascular Coagulopathy and Placental Abruption: A Case Report and Review of the Literature

**DOI:** 10.1155/2019/4305849

**Published:** 2019-04-04

**Authors:** Jordan Myers, Gary Wu, Robert E. Shapiro, Manuel C. Vallejo

**Affiliations:** ^1^Department of Anesthesiology, West Virginia University School of Medicine, Morgantown, WV 26506, USA; ^2^Department of Obstetrics & Gynecology, West Virginia University School of Medicine, Morgantown, WV 26506, USA; ^3^Department of Medical Education, West Virginia University School of Medicine, Morgantown, WV 26506, USA

## Abstract

A 33-year-old primigravida at 32-week gestation was admitted to labor and delivery complaining of severe right upper quadrant pain and worsening coagulopathy. We report the anesthetic and obstetrical management of a complex case of a parturient with a mixed picture of hemolysis, elevated liver enzymes and low platelets who was delivered under general anesthesia further complicated by Disseminated Intravascular Coagulopathy (DIC) and placental abruption.

## 1. Introduction

Preeclampsia in its severe form involves end organ damage requiring multidisciplinary treatment to avoid perinatal mortality [1]. AFLP is a rare disorder defined by microvesicular fatty infiltration of hepatocytes, occurring in the 3^rd^ trimester of pregnancy or early postpartum period [1]. Symptoms develop with accumulation of fatty acid break down products resulting in damage to maternal hepatocytes [2]. Diagnosis can be difficult due to clinical overlap with other common disorders of pregnancy such as cholestasis, viral hepatitis, severe preeclampsia, and HELLP syndrome. AFLP presents with nonspecific findings and varying degrees of severity, further increasing diagnostic difficulty. Prompt delivery of the fetus and patient support are the most important factors for maternal recovery. HELLP syndrome is a complication of pregnancy characterized by hemolysis, elevated liver enzymes, and low platelets (HELLP) [3]. HELLP syndrome, as with any disorder of pregnancy causing liver dysfunction, can result in DIC.

We report the anesthetic and obstetrical management and literature review of a complex case of a parturient with a mixed picture of preeclampsia induced liver dysfunction complicated by Disseminated Intravascular Coagulopathy (DIC) and placental abruption.

## 2. Case Description

**Figure 1:**
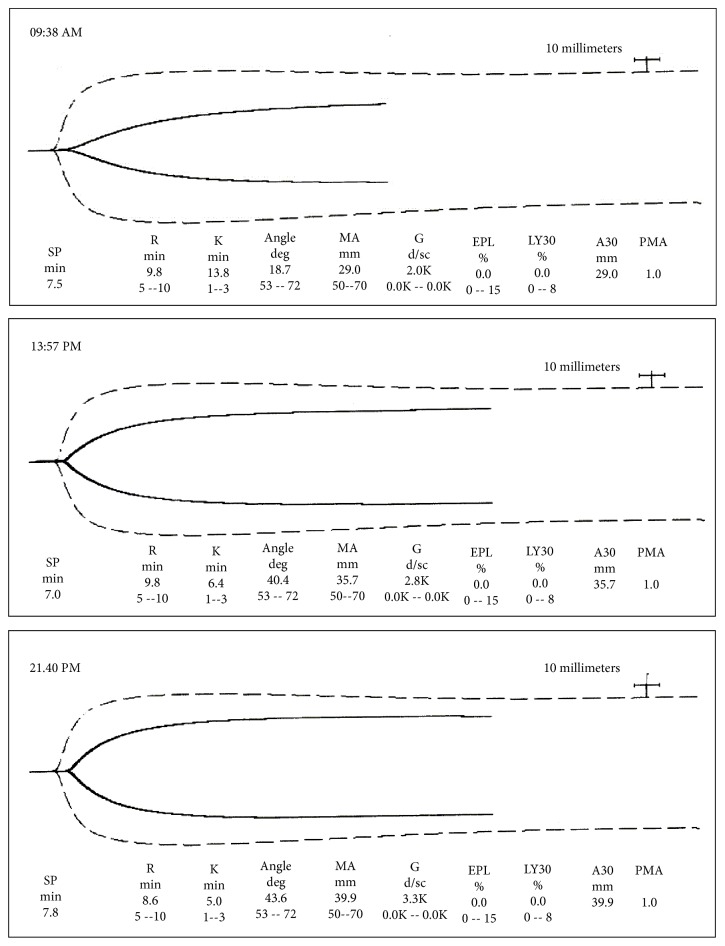
Thromboelastograms (TEGs) before cesarean delivery, after blood replacement therapy, and approximately 12 hours after cesarean delivery. Legend: 09:38 TEG prior to cesarean delivery, note decreased maximum amplitude, elevated K value, and decreased alpha angle; 13:57 TEG after blood replacement therapy, note K value, alpha angle, and maximum amplitude improving but still below normal; 21:40 TEG later that evening, below normal values.

A 33-year-old 78 kg, 155 cm, primigravida at 32-week gestation presented to labor and delivery complaining of severe epigastric chest pain. The patient had been seen in clinic prior to admission for lower extremity edema and headache and was diagnosed with mild preeclampsia. Over the two days prior to admission, she reported shortness of breath and worsening oliguria despite adequate oral intake. Initially, her epigastric pain was thought to be gastroesophageal reflux disorder (GERD) which now worsened to a constant 10/10 pain mostly confined to the right upper abdominal quadrant. Initial laboratory results at 03:52 were unremarkable. More than 3 hours later at 07:21, the platelet count went from 195 K/uL to “*unable to perform count, platelets clumped on slide* Aspartate aminotransferase (AST) increased from 183 u/L to 3180 u/L, and Alanine aminotransferase (ALT) increased from 221 u/L to 3495 u/L (an increase more than 15x admission values). Because of her rapidly worsening clinical picture, the decision was made to proceed with an urgent cesarean delivery. The platelet count was repeated and because of a high clinical suspicion for thrombocytopenia, a thromboelastogram (TEG) was performed, showing features of coagulopathy (Figure 1). Repeat platelet count at 09:28 was again “*unable to perform count, platelets clumped on slide*.” Alarmingly, the AST had increased to 4,059 u/L and ALT to 4,000 u/L. Because of her severe coagulopathy, the patient was not determined to be a candidate for neuraxial anesthesia. Her initial blood pressure on admission was 199/100 mm Hg. Oral labetalol and intravenous hydralazine were given for treatment of her high blood pressure. Intravenous magnesium was started for seizure prophylaxis and treatment of HEELP syndrome.

The urgent cesarean section was delayed until Fresh Frozen Plasma (FFP), cryoprecipitate, platelets, and packed red blood cells (pRBCs) could be available and in the operating room so that blood component therapy could be instituted at the time of the procedure. While waiting for the blood products, a second 18-gauge peripheral intravenous catheter was placed as well as a radial arterial line for assessment of her blood pressure on a beat-to-beat basis, for treatment for hemodynamic instability, and to obtain blood for laboratory analysis.

After blood products were made available in the operating room, intravenous cefazolin 2 gm was given for antibiotic prophylaxis. The patient had taken nothing by mouth since midnight. Induction medications included 200 mg of propofol, 100 mcg of fentanyl, and 160 mg of succinylcholine intravenously under rapid sequence intubation with cricoid pressure, and esmolol (30 mg) to attenuate the hypertensive response to laryngoscopy and intubation. The trachea was intubated on the first attempt with a 7.0 endotracheal tube confirmed by auscultation of bilateral breath sounds and by end tidal CO_2_ monitor detection. Sevoflurane (4%) and 100% oxygen (4 liters) were used for maintenance of anesthesia which was changed to Sevoflurane (1.5%) with 60% nitrous oxide in oxygen after delivery of the baby for prevention of uterine atony. After fetal delivery, 4 mg of midazolam and 2 mg of hydromorphone were administered intravenously over divided doses for additional amnesia and analgesia. Throughout the procedure, the patient's blood pressure trended in the 110-140 mm Hg systolic and 50-80 mm Hg diastolic range.

During hysterotomy, the placenta was noted to be abrupting. A female neonate was delivered weighing 1570 gm with Apgar scores of 5 at 1 minute and 8 at five minutes. Uterine atony was noted with oozing and poor blood clotting. Standard oxytocin dose at West Virginia University Hospital is a 5-unit bolus of oxytocin and 35-unit infusion of oxytocin in 1 liter of lactated ringer's solution. The patient received 250-mcg carboprost intramuscularly and 1000 mcg misoprostol placed intrauterine for treatment of uterine atony. The placental abruption was noted to be small and was able to be controlled upon removal of the placenta. Tranexamic acid (1 gm) was considered but not administered since uterine tone became adequate over several minutes. Intraoperatively, the patient was transfused 2 units of FFP, 2 units of cryoprecipitate, and 1 pack of platelets. Estimated blood loss was over 2.2 liters. After the patient satisfied extubation criteria (train of four > 0.9, sustained head lift for 5-sec, and tidal volume > 5ml/kg), the patient was extubated without complication and able to maintain her airway. The patient recovered in the obstetrical intensive care unit. Bilateral sequential compression devices were placed on both legs and enoxaparin (40 mg SQ QD) was administered for deep venous thromboembolism prophylaxis.

A postoperative TEG was obtained, showing slight improvement in her coagulopathy, consistent with multiorgan system dysfunction (Figure 1). The patient's postoperative course was complicated by worsening coagulopathy, respiratory, and renal dysfunction. The patient required 5 liters of supplemental oxygen via nasal cannula postoperatively for shortness of breath and hypoxia. On postoperative day (POD) 1, arterial blood gas analysis was consistent with respiratory alkalosis (pH=7.42, PCO_2_=30.0, Bicarbonate=21.7, and Base Deficit=3.9). A chest x-ray revealed a left pleural effusion. Pulmonology was consulted for assistance managing her respiratory status and recommended discontinuing magnesium therapy and substituting dilantin due to a concern for magnesium worsening her pulmonary edema. Nephrology was consulted for assistance managing her acute kidney injury as her creatinine increased to 4.69 umol/L on POD-3 from a baseline of 0.78 umol/L on admission. Nephrology recommended continuing treatment for hypertension, avoiding Nonsteroidal Anti-inflammatory Drugs (NSAID's), Angiotensin-Converting-Enzyme Inhibitor (ACEI), Angiotensin-Receptor Blocker (ARB), and contrast dye, and continuing fluid restriction, and hemodialysis was not indicated. Hepatic AST/ALT enzymes which peaked at 4059/4000 u/L at 09:28 the day of surgery continued to steadily improve and fell into the normal range upon hospital discharge. The patient's multiorgan system dysfunction steadily improved over several days, and she was discharged home along with her baby in stable condition on POD-8 with a 1-week follow-up for a blood pressure check and a 6-week obstetric postpartum follow-up.

## 3. Discussion

The diagnosis of preeclampsia, HELLP, and acute fatty liver of pregnancy (AFLP) in the setting of placental abruption all appear to have overlapping criteria and can be difficult to distinguish. Preeclampsia is characterized by new onset hypertension and proteinuria after 20-week gestational age [5]. Without severe features, blood pressure are greater than 140/90 mmHg with proteinuria greater than 300 mg/24-hour period or a point-of-care test of protein-creatinine ratio of 0.3 [5]. Preeclampsia with severe features involves signs and symptoms of end-organ damage: blood pressure greater than 160/100 mmHg, serum creatinine greater than 1.1 mg/dL or twice baseline, elevated liver function tests of twice normal with persistent right upper quadrant pain, severe headaches with or without vision changes, and thrombocytopenia with platelet count less than 100,000/mL [5].

The incidence of AFLP is 1:7000 – 1:15000 pregnancies [2]. In a report by Gregory et al. [6], they discussed 3 cases of AFLP and how variable each case can present. One patient presented with elevated liver function, malaise, and nausea, much like our patient. The two other cases they describe have patients who presented with abdominal pain, pruritus, and nausea. Their patients did not present with elevated blood pressure where our patient did. Stander and Cadden [7] first described AFLP in 1934. Sheehan [8] then described it in 1940 as acute yellow atrophy—where postmortem exams were performed on 400 obstetric patients and 6 were found to have significant liver pathology. Mortality in the past was recorded as high as 75-85% [9]. The mortality has been significantly reduced to 18 to 23% [10]. Today, there is a decrease in mortality from AFLP due to prompt maternal supportive care and delivery of the fetus. In the past, a liver biopsy was indicated for diagnosis of AFLP, leading to delayed management and a risk for bleeding since these patients can have severe coagulopathy. At that time, serology was unavailable for viral hepatitis [11]; and clinically the patients present similarly. Now AFLP has become a diagnosis based on detailed history, laboratory results, and imaging such as liver ultrasound (Table 1). AFLP has nonspecific findings, which are often mistaken for HELLP syndrome, cholestasis of pregnancy, or preeclampsia (Table 2).

HELLP syndrome is a complication of pregnancy characterized by H-hemolysis, EL-elevated liver enzymes, and a LP-low platelet count [3, 5, 12]. HELLP syndrome is a variant of severe pre-eclampsia, occurring in about 0.7% of pregnancies and affects about 15% of women with eclampsia or severe preeclampsia [3, 5, 12]. HELLP usually begins during the last three months of pregnancy or shortly after childbirth [3, 5, 12]. Symptoms may include feeling tired, retaining fluid, headache, nausea, upper right abdominal pain, blurry vision, nosebleeds, and seizures [3, 5, 12]. Complications may include disseminated intravascular coagulation (DIC), placental abruption, acute kidney failure, pulmonary edema, cerebral edema, cerebral hemorrhage, eclampsia, liver hematoma, liver rupture, and death [3, 5, 12]. Diagnostic criteria for HELLP syndrome include microangiopathic hemolytic anemia with schistocytes on peripheral blood smear, thrombocytopenia (platelets <100,000 cells/mL), serum AST > 2 times normal (usually >70 units/L), LDH greater than 600 U/L, and total bilirubin > 1.2 mg/dL [3, 5, 12]. Treatment generally involves delivery of the baby as soon as possible, which is particularly true if the pregnancy is beyond 34 weeks of gestation [3, 5, 12]. Medications should be used to decrease blood pressure and blood transfusions may be required [3, 5, 12]. Additionally, corticosteroids can be given to speed the development of fetal lung maturity [3, 5, 12].

In normal pregnancy, the physiologic increase in glomerular filtration rate (GFR) results in a decrease in serum creatinine, which falls by an average of 0.4ml/dl to a pregnancy range of 0.4 to 0.8 mg/dl [13]. Our case demonstrates the development of acute kidney injury (AKI) because of multisystem organ damage associated with AFLP. The pathogenesis of AKI is still somewhat unclear. In 1974, Finkelstein et al. [14] described “reversible postpartum renal failure” in the setting of hypertension. The glomeruli on renal biopsy showed widespread fibrin deposition of the peritubular capillaries. In 1976, Arias et al. [15] showed fibrin deposition outlining the hepatic sinusoids on needle biopsy in patients with AFLP. This suggests a common mechanism for organ damage, particularly in the kidney and liver. The fact that fibrinogen and its derivatives may be abundant in AFLP patients infers that an abnormality in the coagulation-fibrinolysis system may be an inciting factor.

Diagnosis of AFLP can be made using the Swansea Criteria (Table 1). Six or greater symptoms are necessary for diagnosing AFLP. The sensitivity and specificity of Swansea criteria were 100% and 57% with positive and negative predictive values of 85% and 100% in a study by Goel et al. [4]. Our patient had the following criteria: abdominal pain, ascites (ascites noted on the operative report upon entrance into the abdomen), coagulopathy (elevated PT/PTT), elevated AST/ALT, elevated bilirubin, and encephalopathy.

In comparison to HELLP syndrome, evidence of hepatic insufficiency such as encephalopathy, severely elevated AST/ALT, hypoglycemia, and abnormalities in coagulation studies is more consistent with acute fatty liver of pregnancy [16]. Both HELLP and acute fatty liver of pregnancy can be associated with DIC. Fortunately, treatments of both HELLP syndrome and AFLP are largely the same. Supportive care, delivery of the fetus, and coagulopathy reversal are the mainstays of treatment.

Point of care viscoelastic hemostatic assessment testing is methods of testing the efficiency of blood coagulation and can be beneficial in the correction of coagulation disorders [17]. Both thromboelastography (TEG) and thromboelastometry (TEM) demonstrate the global interaction of platelets in the coagulation cascade (aggregation, clot strengthening, fibrin cross-linking, and fibrinolysis) and can guide transfusion strategy [4]. Point of care viscoelastic hemostatic assessment can supplement the more common tests of blood coagulation including prothrombin time (PT), partial thromboplastin time (aPTT), and the international normalized ratio (INR), [17]. Based on TEG analysis, correction can be provided with specific blood product administration (i.e., reaction time > 5-10 mins [FFP indicated], kinetic time > 1-3 mins [cryoprecipitate indicated], *α*-angle < 53-72 degrees [cryoprecipitate indicated], maximum amplitude < 50-70 mm [platelets and/or DDAVP indicated], and lysis at 30 min > 0-8% [tranexamic acid and/or aminocaproic acid indicated).

In conclusion, preeclampsia, HELLP syndrome, and AFLP are all on the spectrum of end organ disease processes that can result in perinatal mortality. Rapid disease recognition and proactive anesthetic and obstetric multidisciplinary treatment can result in a good outcome for both the mother and baby.

## Figures and Tables

**Table 1:** Swansea Criteria for AFLP [4].

Abdominal Pain

Vomiting

Leukocytosis (> 11 x 10^9^ K/uL)

Polydipsia/Polyuria

Renal Impairment (Creatinine > 150 umol/L)

Ascites or Bright Liver on Hepatic US

Coagulopathy (PT > 14 seconds or PTT > 34 seconds)

Elevated ammonia (> 47 umol/L)

Elevated AST/ALT (> 42 IU/L)

Elevated Bilirubin (> 14 umol/L)

Elevated Urate Level (> 340 umol/L)

Encephalopathy

Hypoglycemia (< 4 mmol/L)

Microvesicular steatosis on liver biopsy

**Table 2:** Patient Clinical Features of AFLP, Preeclampsia, and HELLP Syndrome.

Patient Clinical Features	AFLP	Preeclampsia	HELLP
Hemolysis		X	X

Elevated Liver Enzymes	X	X	X

Low platelets		X	X

Coagulopathy	X	X	X

Hypertension		X	

Headache		X	

Shortness of Breath		X	

Oliguria		X	

Abdominal Pain	X	X	

Renal Impairment	X	X	

Elevated Bilirubin	X	X	

Ascites	X	X	

Encephalopathy	X	X	

HELLP = Hemolysis, Elevated liver enzymes, Low platelets.

## References

[1] Tran T. T., Ahn J., Reau N. S. (2016). ACG clinical guideline: liver disease and pregnancy. *American Journal of Gastroenterology*.

[2] Castro M.-A., Fassett M. J., Reynolds T. B., Shaw K. J., Goodwin T. M. (1999). Reversible peripartum liver failure: a new perspective on the diagnosis, treatment, and cause of acute fatty liver of pregnancy, based on 28 consecutive cases. *American Journal of Obstetrics & Gynecology*.

[3] Haram K., Svendsen E., Abildgaard U. (2009). The HELLP syndrome: Clinical issues and management. a review. *BMC Pregnancy and Childbirth*.

[4] Goel A., Ramakrishna B., Zachariah U. (2011). How accurate are the Swansea criteria to diagnose acute fatty liver of pregnancy in predicting hepatic microvesicular steatosis?. *Gut*.

[5] Espinoza J., Vidaeff A., Pettker C. (2019). ACOG practice bulletin no. 202: gestational hypertension and preeclampsia. *Obstetrics & Gynecology*.

[6] Gregory T., Hughes S., Coleman M., De Silva A. (2007). Acute fatty liver of pregnancy; three cases and discussion of analgesia and anesthesia. *International Journal of Obstetric Anesthesia*.

[7] Stander H. J., Cadden B. S. (1934). Acute yellow atrophy of the liver in pregnancy. *American Journal of Obstetrics & Gynecology*.

[8] Sheehan H. L. (1940). The pathology of acute yellow atrophy and delayed chloroform poisoning. *The Journal of Obstetrics and Gynaecology of the British Empire*.

[9] Ko H., Yoshida E. (2006). Acute fatty liver of pregnancy. *Canadian Journal of Gastroenterology & Hepatology*.

[10] Kaplan M. M. (1985). Acute fatty liver of pregnancy. *The New England Journal of Medicine*.

[11] Ober W. B., LeCompte P. M. (1955). Acute fatty metamorphosis of the liver associated with pregnancy; a distinctive lesion. *American Journal of Medicine*.

[12] Aloizos S., Seretis C., Liakos N. (2013). HELLP syndrome: understanding and management of a pregnancy-specific disease. *Journal of Obstetrics & Gynaecology*.

[13] Fischer M. J. (2007). Chronic kidney disease and pregnancy: maternal and fetal outcomes. *Advances in Chronic Kidney Disease*.

[14] Finkelstein F. O., Kashgarian M., Hayslett J. P. (1974). Clinical spectrum of postpartum renal failure. *American Journal of Medicine*.

[15] Arias F., Mancilla-Jimenez R. (1976). Hepatic fibrinogen deposits in preeclampsia - immunofluorescent evidence. *The New England Journal of Medicine*.

[16] Knight M., Nelson-Piercy C., Kurinczuk J. J., Spark P., Brocklehurst P. (2008). A prospective national study of acute fatty liver of pregnancy in the UK. *Gut*.

[17] da Luz L. T., Nascimento B., Rizoli S. (2013). Thrombelastography (TEG®): practical considerations on its clinical use in trauma resuscitation. *Scandinavian Journal of Trauma, Resuscitation and Emergency Medicine*.

